# Towards a European Framework to Monitor Infectious Diseases among Migrant Populations: Design and Applicability

**DOI:** 10.3390/ijerph120911640

**Published:** 2015-09-17

**Authors:** Flavia Riccardo, Maria Grazia Dente, Tommi Kärki, Massimo Fabiani, Christian Napoli, Antonio Chiarenza, Paolo Giorgi Rossi, Cesar Velasco Munoz, Teymur Noori, Silvia Declich

**Affiliations:** 1 National Centre for Epidemiology, Surveillance and Health Promotion, National Institute of Health (Istituto Superiore di Sanità, ISS), viale Regina Elena, 299-00161 Rome, Italy; E-Mails: mariagrazia.dente@iss.it (MGD); tommi.karki@iss.it (T.K.); massimo.fabiani@iss.it (M.F.); christian.napoli@iss.it (C.N.); silvia.declich@iss.it (S.D.); 2 European Programme for Intervention Epidemiology Training (EPIET), European Centre for disease Prevention and Control (ECDC), Tomtebodavägen 11a, 171 83 Stockholm, Sweden; 3 Research and Innovation Unit AUSL (Azienda Unità Sanitaria Locale) Reggio Emilia, Reggio Emilia 42122, Italy; E-Mail: antonio.chiarenza@ausl.re.it; 4 Interinstitutional Epidemiology Unit, AUSL (Azienda Unità Sanitaria Locale) Reggio Emilia, Reggio Emilia 42122, Italy; E-Mail: paolo.giorgirossi@ausl.re.it; 5 European Centre for Disease Prevention and Control (ECDC), Tomtebodavägen 11a, 171 83 Stockholm, Sweden; E-Mails: cesar.velascomunoz@ecdc.europa.eu (C.V.M.); teymur.noori@ecdc.europa.eu (T.N.)

**Keywords:** transients and migrants, European Union, epidemiology, communicable diseases, data collection

## Abstract

There are limitations in our capacity to interpret point estimates and trends of infectious diseases occurring among diverse migrant populations living in the European Union/European Economic Area (EU/EEA). The aim of this study was to design a data collection framework that could capture information on factors associated with increased risk to infectious diseases in migrant populations in the EU/EEA. The authors defined factors associated with increased risk according to a multi-dimensional framework and performed a systematic literature review in order to identify whether those factors well reflected the reported risk factors for infectious disease in these populations. Following this, the feasibility of applying this framework to relevant available EU/EEA data sources was assessed. The proposed multidimensional framework is well suited to capture the complexity and concurrence of these risk factors and in principle applicable in the EU/EEA. The authors conclude that adopting a multi-dimensional framework to monitor infectious diseases could favor the disaggregated collection and analysis of migrant health data.

## 1. Introduction

After the end of the World War II, Europe shifted from being a major source of emigration to become a major destination for human migration [1]. There has been a steady increase in the number of international migrants living in Europe from the 1960s, when they were around 3.5% of the total population [1]. This trend accelerated in the 1990s, when countries in Southern Europe also became the destination of large migration flows [2]. As a consequence, there are different migration patterns across the European region, with some European Union (EU) countries only recently observing increases in the number of economic immigrants and asylum seekers, and others where migrants and their descendants have, over time, acquired a demographic and social stratification.

Since 2011, geopolitical instability in the Middle East and North Africa has been contributing to exceptional inflows of migrants to Europe across the Mediterranean Sea [2], which has become the deadliest migration route worldwide [3]. The United Nations High Commissioner for Refugees (UNHCR) recorded consistent increases in the number of people seeking refugee status in Europe, and has linked this mainly to conflict/instability in Syria from 2012 [4] and, more recently, also in Iraq, Afghanistan, and Eritrea [5]. In 2014, 38 European countries recorded 264,000 asylum applications, a 24% increase compared to the previous year [5].

Migration is now recognized as one of the key components of population change in Europe [6]. As of 1 January 2013, 20.4 million people with a citizenship of a non-member country resided in countries of the EU, representing 4.1% of the EU population, and 13.7 million people living in an EU country were citizens of another EU country [7]. As a consequence of all the mentioned evolving and complex human mobility patterns involving the European region, the migrant population within the EU is extremely diverse in terms of country of origin, reasons of migration, lengths of stay and socio-economic backgrounds [2].

Recent evidence on the health status of migrants arriving in the EU/European Economic Area (EEA) (the European Economic Area includes all the European Union Member States, Iceland, Liechtenstein, and Norway) [8] has been reassuring, as in the case of the results of syndromic surveillance system set up in Italy [2,9]. This system did not highlight health emergencies in relation to exceptional migration inflows between 2011 and 2013. The lack of relevant health issues associated with recent migration flows, supports the “*healthy migrant effect*” concept [10,11,12]. According to this hypothesis, “*health selection has a positive effect on migrants’ health outcomes, especially in the first years after migration*” [10]. Nevertheless, migrants can be at risk of developing disease, including infectious diseases, due to living conditions or other disparities [13]. For this reason, addressing determinants of health and inequalities in access to care are essential components of any program aiming to improve migrant health in host countries [14]. Notwithstanding, there is some evidence that migration-driven demographic changes may contribute to the burden of some infectious diseases, such as tuberculosis (TB) and hepatitis B, in countries receiving immigrants from highly endemic areas [15,16], by increasing the total number of cases observed. Even though increased disease transmission from migrant to host communities in receiving countries has not been widely documented [17], some studies suggest links between migration and the spread, or the risk for reintroduction, of infectious diseases in Europe [18,19,20,21,22]. For this reason, there is still uncertainty on the contribution of migration to the burden of infectious disease in the European Union/European Economic Area (EU/EEA).

Health service-based surveillance is most commonly used to monitor the epidemiology of infectious diseases. Among EU/EEA Member States, infectious diseases of public health relevance are notifiable in application to an EU legal framework for communicable disease surveillance [23], which includes the EU Decision No. 2119/98/EC [24] and the subsequent EU Decision No 1082/2013/EU on serious cross-border threats to health [25]. This legal framework defines a common standard for epidemiological surveillance in the EU. In 2014, an analysis of the burden of infectious diseases among migrant populations in the EU/EEA based on this surveillance data, was published by the European Centre for Disease Prevention and Control (ECDC) [26]. ECDC concluded that there is evidence showing that migrants carry a disproportionate burden of TB, HIV and chronic hepatitis B. However, these authors also pointed out that drawing overall conclusions about infectious disease burden among migrants was challenging due to limitations in the data and differences in reporting between countries. In this study, infectious disease data was stratified mainly according to the variable “Country of Birth”, in line with the current ECDC definition of migrant as anyone foreign-born. This stratification alone does not distinguish between intra EU mobility and migration or take into account the mentioned diversity of migrant populations in the EU/EEA. It also fails to consider the length of stay in the EU/EEA and prior migration trajectories. This limits the capacity to interpret point estimates and trends of cases of infectious diseases occurring among foreign-born people in the EU/EEA.

Having in place multiple data collection domains allowing us to stratify cases of an infectious disease among migrant populations according to factors associated with an increased risk of contracting it, would improve our understanding of what population groups are most affected and increase our ability to interpret time trends. This in turn could facilitate the formulation of targeted plans for public health action. However, we currently lack this stratified data [27,28,29] and there is no evidence on what data collection framework could capture information on factors associated with increased risk to infectious diseases in migrant populations in the EU/EEA.

We conducted a systematic review of scientific literature focusing on infectious diseases occurring among migrant populations in order to identify how multiple data collection domains could be used in monitoring infectious diseases. We analyzed how risk factors for infectious diseases were described, and classified them according to a multi-dimensional monitoring framework taking into account migration-specific, behavioral and socioeconomic factors, alongside demographic characteristics. We then analyzed existing relevant data sources in the EU/EEA in order to assess the applicability of this framework.

## 2. Methods

### 2.1. Systematic Literature Review

#### 2.1.1. Inclusion Criteria

We considered scientific articles for inclusion if they: (i) were case reports and case series studies, descriptive and analytic epidemiological studies, literature reviews, or qualitative medical socio-anthropologic studies; (ii) were published between 2010 and 2013; (iii) were published in English or French; and (iv) included data on infectious diseases in migrant populations.

Studies were included regardless of the country of publication.

#### 2.1.2. Search Strategy

We defined two search strings (Table 1) and searched articles in PubMED. One reviewer conducted a first screening for relevance on title and abstracts. Following this, potentially relevant articles identified were downloaded and reviewed in full text by the same reviewer.

**Table 1:** Search strategy.

Key Words	Search String	Details	DB
Migrant + infectious	1	((migrant[Title/Abstract]) AND infectious[Title/Abstract])	Pubmed
Infectious diseases in newly arrived migrants or Asylum seekers or irregular migrants	2	((((((((((((((((((((infection) OR infectious) OR outbreak) OR contagious) OR tuberculosis) OR TB) OR HIV) OR hepatitis) OR HBV) OR HCV) OR poliomyelitis) OR meningitis) OR gonorrhea) OR syphilis) OR malaria) OR chagas) OR measles) OR rubella) AND newly arrived migrants) OR asylum seekers) OR irregular migrants Filters: From 01/01/2010 to 31/12/2013, Humans	Pubmed

#### 2.1.3. Data Extraction and Analysis

We collected information on study design, study size, geo-representativity of study results (international/national/subnational/service based) and on the infectious disease(s) described (hereby infectious disease conditions).

For each infectious disease condition, the reviewer examined what factors were reported to increase the risk of the population studied, either in the study results or in the discussion quoting additional scientific evidence.

**Figure 1:**
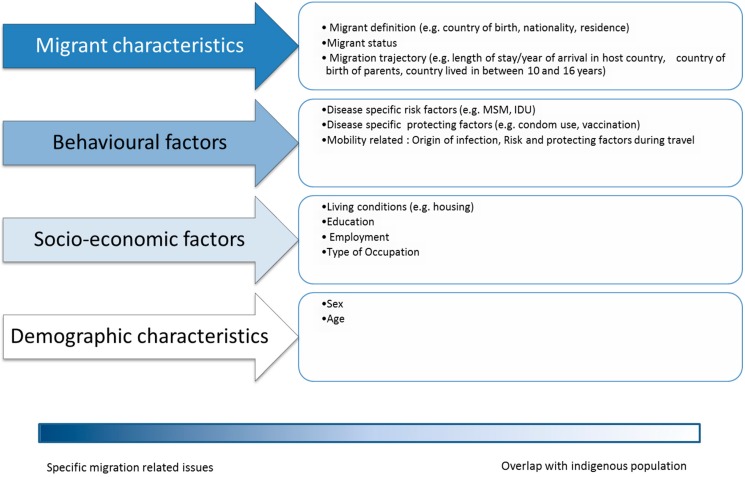
Factors/characteristics identifying the four data collection domains and examples of type of variables under each domain.

In order to identify variables needed to describe those risk factors, we divided them according to four data collection domains (Figure 1):

1. “migration characteristics”, *i.e.*, including variables/factors uniquely associated with being a migrant. These included: migrant legal status, migration trajectory (country of origin/travel route) and access to health care in the host country. For the purpose of this study the term “Variable” and “Factor” are used as synonyms. The first term is preferred to describe the type of information recorded in databases (either to be included in the hypothetical data collection framework or currently included in existing meta-datasets). The second is used when describing information presented in published studies (not datasets);
2. “behavioral factors”, *i.e.*, including variables/factors that could be more frequently associated with migrant communities but not exclusively. These included: disease specific risk factors and mobility (*i.e.*, travel) related factors;
3. “socioeconomic factors”, *i.e.*, including variables/factors that could be more frequently associated with migrant communities but not exclusively. These included: poverty (living conditions/employment), education and (lack of) occupation;
4. “demographic characteristics”, *i.e.*, including variables/factors completely uninfluenced by being a migrant: such as age and/or sex when those were identified as a risk factor.

We performed an infectious disease condition specific frequency analysis of all the factors/variables mentioned as a risk for acquiring an infectious disease.

### 2.2. Feasibility of Applying the Identified Four Data Collection Domains

We examined the variables currently collected by EU/EEA Member States by reviewing The European Surveillance System (TESSy) metadataset. We classified the existing variables according to the four domains, and we examined the feasibility of aligning this data with denominators of migrant population estimates for the EU/EEA by examining the public websites and the data published by the three existing official sources of data: the statistical office of the European Union (Eurostat), the United Nations Population Division Department of Economic and Social Affairs (UN-DESA) and the UNHCR. For each of them we analyzed the type of variables collected and, where possible, how they were defined. The frequency of publication of updated information and time references of the population estimates were also assessed.

## 3. Results

### 3.1. Systematic Literature Review

**Figure 2:**
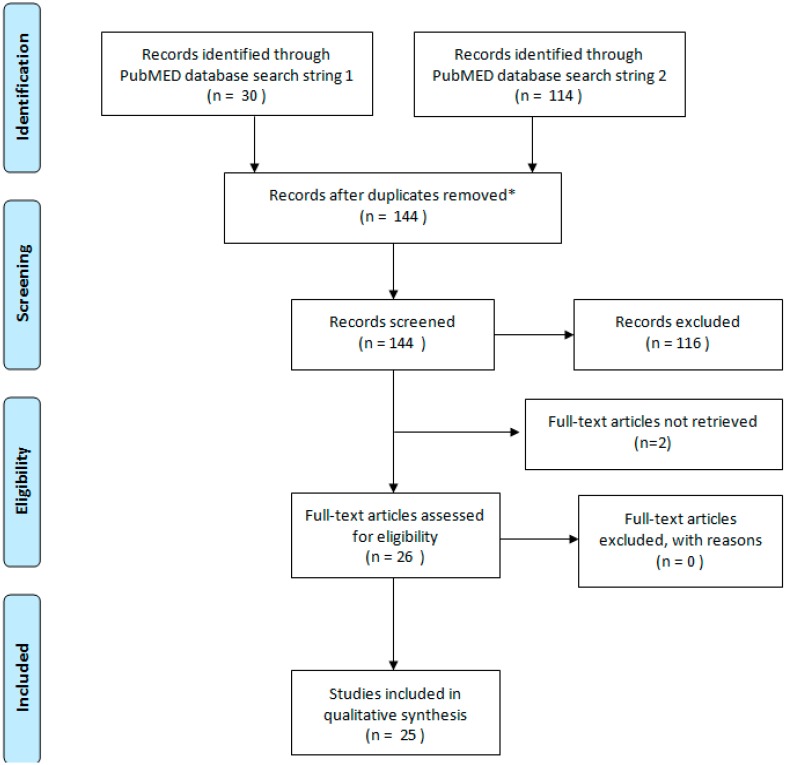
Flow Diagram of the systematic review (modified from PRISMA (Preferred Reporting Items for Systematic Reviews and Meta-Analyses) statement [30]). * No duplicates were found.

We identified 144 potentially relevant articles, 30 using search string 1 and 114 using search string 2 (Figure 2). Ninety-two percent were published between 2011 and 2013.

We assessed titles and abstracts, 116 articles were excluded because they did not match the inclusion criteria. Twenty-eight publications met the inclusion criteria, originating from 27 studies (one study was published in two papers), 12 were identified through search string 1 and 16 through search string 2. Twenty-five papers were in English and three in French, 22 concerned EU countries, four included data from non-EU countries with long migration histories (United States and Israel) and 23 were published between 2012 and 2013. Two articles were not available. The two articles describing the same study were both assessed in full text. Results of those two papers were reported only once in the analysis, which is, therefore, based on 25 studies (Table 2).

As shown in Table 2, the 25 selected studies included 10 case series (of which two with less than 10 observations), four cohort studies, eight cross sectional studies, two social-anthropologic qualitative studies and one literature review. Almost half of the studies presented data obtained from health care services (11 studies, 44%), three presented subnational data from an intermediate administrative level (12%), nine presented national data (36%), and two articles presented international/multinational data (8%).

Fourteen studies focused on more than one infectious disease condition (Table 2). Overall, 14 different infectious disease conditions were reported 35 times in the 25 studies assessed. TB, addressed in 12 studies, was the most frequently addressed condition, followed by HIV that was addressed in five studies.

It was not possible to separately analyze infectious disease conditions in six studies because pathogens were either not mentioned or not studied individually. Those articles focused on the following conditions occurring among migrant populations: (1) “Respiratory and gastrointestinal infections”; (2) “All deaths due to infectious diseases”; (3) “HIV, Malaria, hepatitis, worm infections, insufficiently treated infectious diseases, missed vaccinations”; (4) “Chest infections, HIV, HBV, HCV, Syphilis, TB”; (5) “HIV, Skin problems, Breathing problems, muscle-skeletal problems”; and (6) “Chronic infectious diseases—HIV, HBV, HCV”.

As all those studies addressed at least one potential or declared infectious disease condition, they were classified as focusing on “multiple conditions”. “Multiple conditions” was then analyzed as a separate infectious disease condition.

All the data collection domains included factors that were considered, at least once, as a possible risk in the studies examined.

**Table 2:** Studies included in the systematic review (25 studies in 26 published articles).

	Authors	Language	Country	Focus	Type of Study	Geo-representativity	Study Size
**Search String 1**	Simon (2013) [31]	English	France	Severe cutaneous infections	Case series	Service based	7 hospitalized patients
	Wagner (2013) [32]	English	United Kingdom	Multiple conditions	Case series from surveillance data	National	TB > 6000 per year; HIV > 6000; Malaria between 1300 and 2000 per year; enteric fevers 400–500 per year
	Barnett (2013) [17]	English	United States	Multiple conditions	Case series from surveillance data	Service based	7792 systematic migrant protocol screening records
	Zammarchi (2012) [33]	English	Italy	Syphilis	Case series	Service based	187 records of pregnancy of which 143 followed to the end
	Rosales (2012) [34]	English	US And MEXICO	Seasonal farm workers	Cross sectional study	Subnational (intermediate level)	Survey on 233 *jornaleros* migrant workers
	Jaeger (2012) [35]	English	Switzerland	Children	Systematic literature review	National	Studies quoted included a study on TB on 234 children and HAV surveillance data.
	Kamper-Jørgensen (2012) [36]	English	Denmark	TB	Case series from surveillance data	National	4631 genotyped TB cases
	Boulogne (2012) [28]	English	France	Mortality	Case series from mortality data	National	Complete mortality data Mainland France 2004–2007 (251, 665 foreign born)
	Kehr (2012) [27]	English	France and Germany	TB	Social/anthropologic qualitative study	International	NA
	Norredam (2012) [37]	English	Denmark	Mortality	Cohort study	National	56,273 refugees and immigrants and 225,090 Danish controls
	Tafuri (2011) [38]	English	Italy	TB	Cross sectional study	Service based	982 asylum seekers in a reception center (screening)
	Ott (2010) [39]	English	Germany, Israel	Mortality	Cohort study	International	Immigrants from former soviet union states: 34,393 randomly selected in North Rhine state Germany and 528,848 in Israel
**Search String 2**	Suurmond (2013) [40]	English	Netherlands	Multiple conditions	Social/anthropologic qualitative study	National	A purposive sample (non-probabilistic) of nurse practitioners and PH physicians in 50 asylum seeker centres (6 clusters)
	Stoffels (2013) [41]	English	Belgium	TB	Cohort study	National	174 MDR TB patients from National Tuberculosis register
	Kan (2013) [42]	English	Sweden	TB	Case series service based	Service based	415 consecutive patients in a Swedish TB clinic
	Nyiri (2012) [43]	English	United Kingdom	Multiple conditions	Case series service based	Service based	First 112 patients who completed questionnaire seen at a refugee clinic London
	Sarivalasis (2012) [44]	English	Switzerland	TB	Cross sectional study	Subnational (intermediate level)	Interview and testing of 393 newly arrived asylum seekers in two Swiss hosting centers
	Chai (2013) [45]	English	United States	Chronic infectious diseases (ID)	Cohort study	Subnational (intermediate level)	630 asylees and 151 refugees of the District of Columbia
	Fenner (2012) [46]	English	Switzerland	chronic ID	Cross sectional study	National	381 TB patients (of whom 103 HIV co-infected)
	Takla (2012) [47]	English	Germany	Measles	Case series	Service based	Eight cases of measles in an asylum seekers’ shelter hosting 427 residents
	De Valliere (2011) [48]	English	Switzerland	Varicella	Case series	Service based	16 cases of varicella in a housing facility for 125 asylum seekers
	Redman (2011) [49]	English	United Kingdom	Multiple conditions	Cross sectional study	Service based	Survey among 30 asylum seekers in an initial accommodation center in Wales
	Dudareva (2011) [50]	English	Germany	S. Aureus MRSA	Cross sectional study	Service based	Convenience sample of 232 of 427 residents in an asylum seekers center
	Wickramage (2013) [51,52]	English	Sri Lanka	Malaria	Cross sectional study	National	287 screened returnees (first 6 months of following study); 534 irregular returnees screened for Malaria (32 positive P falciparum)
	Kaoutar (2012) [53]	French	France	Chronic ID	Cross sectional study	Service based	Survey among 536 immigrant patients in a facilitated access outpatient clinic

The factors most frequently cited were included in domain 1 “migration characteristics” (Table 3). For 33 (94.3%) of the 35 conditions, “migrant status”, “migrant trajectory” and/or “access to healthcare” were mentioned by authors as a possible risk factor. Among those, the most reported factor was “migrant status”, *i.e.*, the legal status of the migrants (e.g., refugees, asylum seekers, irregular migrants, seasonal migrant workers) that was mentioned in studies for 28 of the 35 conditions reported. Migrant status has been directly associated with an increased risk of infectious diseases: “*Asylum seekers and refugees are a small portion of the total immigrants in Italy, but represent a sub-group at particularly high risk for TB.”*(Tafuri S. *et al.* 2011) [38]. Migrant status has also been linked to a number of factors that could contribute to this increased risk: “*… refugees and immigrants tend to be less educated, have lower incomes and lower job rates, which may be linked to substandard living environments”* (Norredam M. *et al*. 2012) [37]; “*The vulnerable position of asylum seekers is related to their flight: or more specifically to health conditions before or during their flight and during their asylum procedure”.* (Suurmond J. *et al.* 2013) [40].

The migration trajectory (*i.e.*, the country of origin or the migration route/type of travel) and access to the host country healthcare system were mentioned for respectively 17 and 15 of the 35 infectious disease conditions reported. Country of origin was described as a specific risk factor for acquiring an infection: the“ *analysis* [of enhanced surveillance data for TB, HIV and malaria among migrant populations in the UK] *highlight the importance of region of birth as a risk factor for infection*” (Wagner K.S. *et al*. 2013) [32] or of acquiring an infection at an earlier age “*In general, Nordic* [TB] *cases, excluding Greenlandic cases, were approximately twice as old as African cases at the time of diagnosis”* (Kamper-Jørgensen *et al*. 2012) [36]. Simon F. and co-authors [31], in a 2013 case series, describe travel trajectory as a specific risk factor: “*Infections were favoured by the extreme tropical conditions on the boat, skin maceration in the salty water, starvation and promiscuity. The severe fasciitis in young adults probably results from skin colonization and subsequent infection with staphylococcus and/or streptococcus, underlying muscle traumas, the delay in adequate care, all of which were present during this long drifting period in the Red Sea*”. Also in the context of migration trajectory, length of stay was identified as a protective factor against dying of an infectious disease among males in a cohort study of former soviet union migrants residing in Germany and Israel “*Looking at* *both migrant cohorts together, all cause mortality in males significantly decreased with increasing duration of residence […] for infectious diseases, males with the longest duration of stay had only half the rate* [relative risk] *compared with those in the initial years following arrival.”* (Ott J.J. *et al*. 2010) [39].

**Table 3:** Factors linked with increased risk for specific Infectious Disease Condition groups in migrants.

Infectious Disease Condition (IDC) Group	N. of Studies Addressing the IDC Group	Data Collection Domain 1			Data Collection Domain 2		Data Collection Domain 3			Data Collection Domain 4
		“Migration Characteristics”			“Behavioral Characteristics”		“Socioeconomic Factors”			“Demographic Characteristics”
		Migrant Status	Migration Trajectory	Access to Health Care	Disease Specific Risk Factors	Mobility Related Factors	Poverty	Education	Occupation	Age/Sex
HAV	1	0	0	0	0	1	0	0	0	0
HBV	2	2	0	0	0	0	0	0	0	0
HIV	4	3	2	3	1	0	2	1	0	1
Infectious Hepatitis (not specified)	1	1	0	1	0	0	1	1	0	0
Intestinal Parasites	1	1	0	0	0	0	0	0	0	0
Malaria	2	1	2	1	0	1	0	0	0	0
Measles	1	1	0	0	1	0	0	0	0	0
Multiple conditions	6	6	4	6	0	0	3	2	1	2
Skin infections	2	2	1	0	2	0	0	0	0	0
STD	1	0	1	1	1	0	0	0	0	0
TB	11	9	6	3	4	1	5	1	0	3
TB/HIV	1	1	1	0	0	0	0	0	0	0
Typhoid	1	0	0	0	0	1	0	0	0	0
Varicella	1	1	0	0	1	0	0	0	0	0
Total	35	28	17	15	10	4	11	5	1	6
N. of studies per IDC reporting at least one factor in the domain		33			14		11			6

Access to health care was identified by Boulogne R. and co-authors [28] among several factors possibly related to infectious diseases mortality among migrant populations in an analysis of mortality data of 251,665 foreign born people in Mainland France between 2004 and 2007 “… *the higher mortality due to infectious diseases, and especially AIDS, might be related to poor access to the healthcare system, strongly linked to socioeconomic position. Indeed, the higher mortality by AIDS could be explained by a late access to care and screening (InVS, 2006) and difficulties in adherence to the antiretroviral treatment. Migrant populations, especially those coming from Sub-Saharan Africa and irregular migrants, are specifically affected by such problems.*” This concept was reiterated, for example by Norredam M. and co-authors [37]: “*Restrictions to legal access to healthcare for certain migrant groups may increase disease severity.”* It is worthy to mention that barriers were also identified in contexts where access entitlement is granted: “*every asylum seeker is entitled to free National Health Service (NHS) care […] However barriers to appropriate health care exist including communication problems and issues of fear and mistrust”* (Redman E.A. *et al.* 2011) [49].

The second group of factors identified was included in domain 2 “behavioral factors”. Fourteen (40%) of the 35 infectious disease conditions described either disease specific risk factors and/or mobility related factors as a risk for infectious diseases. Living in overcrowded settings as a risk factor for human-to-human transmitted infections was the main “Disease specific risk factor” mentioned. This has been linked to the reception of migrants in dedicated institutions or shelters within host countries: “*However difficult travel conditions, housing placement in institutions with very large communities, frequent overcrowded conditions and a lack of close supervision of residents of the reception centres are risk factors that aid the spread of TB and other infectious diseases”* (Tafuri S. *et al*. 2011) [38]. Specific challenges in outbreak containment have also been identified in these settings “*During the past years, outbreaks of varicella and measles have been reported in migrant populations or in asylum shelters in Germany. […] outbreaks in such settings impose unique challenges […] involving language barriers, cultural differences, potentially dismissive habitus of residents towards public authorities due to previous negative experiences, large family sizes and unavailability of medical or vaccination records.”* (Takla A. *et al*. 2012) [47].

Mobility was described as a factor contributing to the risk of acquiring an infection: “*For both malaria and enteric fevers, travellers visiting friends and relatives comprise the main risk group of those travelling abroad from the UK*...” and of not completing treatment “…*The most common reason for migrants not completing TB treatment was loss to follow-up […] most of these cases returned to their countries of origin following diagnosis; mostly low-income countries in South Asia and Sub-Saharan Africa*” (Wagner K.S., 2013) [32].

The third group of factors identified was included in domain 3 “socioeconomic factors”. For 11 (31.4%) of the 35 infectious disease conditions reported in the studies, “poverty (living conditions/employment)” and/or “education” and/or “occupation” were mentioned as a risk factor. Among those, the most reported factor was “poverty (living conditions/employment)” that was mentioned in studies for 11 of the 35 conditions reported. Rosales C. and co-authors [34] in a 2012 study of health hazards among migrant and seasonal farmworkers in the US-Mexico border region conclude “*It is clear that a combination of the stress of daily working and living environment, low educational levels, high poverty rates and the lack of access to preventive and routine health care are issues faced by both populations* [migrant farmworkers in the US and Mexico]. *Poor diet is also an issue …*”. Quoting additional studies Norredam M. *et al*. [37] state “*… substandard housing, overcrowding and poor sanitation may contribute to increased risks of infectious diseases among migrants”*.

Finally, for 6 of the 35 conditions addressed in the studies (17.1%) age and/or sex were mentioned as a risk factor (classified under domain 4 “demographic characteristics”). A case series on 415 consecutive patients in a Swedish TB clinic found that “*Among male patients, Somali origin did not reach statistical significance* […] *but there was a significant association with non-completion* [of treatment] *among females”*(Kan B. *et al.* 2013) [43]. Boulogne’s mortality study [28] identified age and sex among the factors diversifying locally-born and foreign-born mortality in France: “*The figures varied by age (higher foreign-born mortality for the young; lower mortality for migrants aged 15-64 years), gender (women more frequently had higher relative mortality), country of birth […] and cause of death (migrant mortality was higher overall for deaths caused by infectious diseases and diabetes …”.* One article (Sarivalasis *et al.* 2012) [44] indicated also “marital status” as a co-factor related to increased risk to latent TB infection. This factor could be included among those in domain 4 “demographic characteristics”.

Twenty-six (74%) of all infectious disease conditions addressed were described using more than one domain in the same study. All four domains were cited by one study on TB, three domains were cited in four studies on TB (of which two on latent TB infection), in one on HIV (specifically on AIDS deaths) and on one reporting multiple conditions (that included chest infections, HIV, TB, HCV, HBV and Syphilis). Two domains were cited by authors reporting on HIV (two studies), infectious hepatitis (one study), malaria (one study), measles (one study), skin infections (two studies), sexually transmitted diseases (one study), TB (two studies), Chickenpox (one study) and multiple conditions (three studies).

### 3.2. Feasibility of Applying the Identified Four Data Collection Domains (DCD) to the Data Collected According to/EU Legislation

#### 3.2.1. Data Collection Domain 1: Migration Characteristics

“Country of Birth” or “Country of Nationality” (defined as citizenship) are the only variables reported by EU/EEA Member States that have been used to define a case as a “migrant” in relation to the reporting country. “Region of Origin” is also available but by definition reflects “Country of Birth”. It should be noted that in the surveillance of measles and rubella, at EU level, the variables “Country of Birth” and “Country of Nationality” are not requested.

#### 3.2.2. Data Collection Domain 2: Behavioral Factors

There is a dearth of currently collected data that can be used to describe behavioral factors. Some information on individual attitudes and behavior can be inferred from the variable “Route of infection”, while some information on mobility can be gathered from the variable “Imported Case/Probable Country of Infection”.

The variable “Route of infection” is currently included in the surveillance of HIV, HBV, and HCV. It describes the most probable route of transmission and indicates the exposures that most likely led to infection. For this reason it could be used as a proxy variable to stratify by different risk groups.

The variable “Imported case” is the only information linked to mobility jointly collected by EU/EEA Member States. It is recorded when health care workers make a judgment about whether a case was likely to have been acquired in the reporting country (*i.e.*, an indigenous case) or in another country following recent travel (*i.e.*, an imported case). The variable “Probable country of infection” is recorded if a case is notified as “imported”. The variable includes each country visited during the incubation period of the disease.

#### 3.2.3. Data Collection Domain 3: Socio-economic Factors

Socio-economic variables are not collected by EU/EEA Member States reporting infectious diseases in application to EU legislation.

#### 3.2.4. Data Collection Domain 4: Demographic Characteristics

This domain includes demographic data that is unrelated to migration status: age and sex. Within the EU/EEA surveillance system, age is collected in months if the age of a case is below two years. Otherwise, this variable is collected in years. Sex is collected through a variable called “gender” that allows four alternatives: female, male, other (e.g., transgender), or unknown.

#### 3.2.5. Denominators

At EU/EEA level, official denominators on regular migrants present in the EU/EEA are available through the statistical office of the European Union, Eurostat [54] and the United Nations Population Division Department of Economic and Social Affairs [55]. The numbers of irregular migrants are also estimated but are unlikely to be reliable. Furthermore, data published by Eurostat and UN-DESA only identify as migrants people with a history of migration of at least one year. Data on refugees, asylum-seekers, returned refugees, internally displaced persons (IDPs) protected/assisted by the UNHCR, returned IDPs, stateless persons in over 180 countries, are published by the UNHCR [56].

Eurostat publishes a number of datasets including: migration and citizenship data, data on asylum applications and decisions, acquisition of citizenship, international migration flows and distributions by age, sex, educational attainment and employment status by broad group citizenship and country of birth. These datasets are updated every year. As of 2015, estimates provided by UN DESA are available only for the years 1990, 2000, 2010 and 2013. The UNHCR database currently contains data from the year 2000 up to 2013. It includes data on its population of concern by status, location of residence or origin, sex and age. Concerning asylum seekers, UNHCR provides information on asylum applications per year and on the refugee status determination process.

## 4. Discussion

Infectious disease risk factors among migrant populations have been attributed, in the studies we reviewed, to migration-specific risks, such as country of origin and migration trajectory, and to migration specific health access barriers, that differ according to the migration status. Vulnerabilities have also been linked to behavioral and socio-economic dimensions that are more frequent among migrants, but not exclusive. We also found that most (74%) of the infectious disease conditions were described according to several concurrent risk factors across different domains. This evidence supports the need to collect data on infectious diseases among migrant populations also taking those factors into account. The application of a multi-dimensional data collection framework, able to support a stratified data analysis according to recognized risk factors, could improve our ability to interpret point estimates and trends. An improved interpretation of infectious disease data is needed to identify sub-groups of the migrant population at higher/lower risk of disease and changes in case distribution over time. This is also needed to clarify current uncertainties on the burden of infectious disease among migrant populations in the EU/EEA and to better target public health action. Our analysis showed that the data collection domains we used were well suited to address the major known risk factors for infectious diseases among migrant populations (Table 3).

Among variables collected in statutory surveillance of infectious diseases among EU/EEA countries, “Country of Birth” and, with lesser frequency, “Country of Nationality” have been used to define a case as “migrant” during data analysis [26]. There are advantages in the use of these variables. Foremost they are simple and widely collected in EU/EEA infectious disease surveillance systems, and they are reported under EC legislation by all Member States. It is also possible, at least in theory, to match “Country of Birth” with denominators (by age and sex) provided every year by Eurostat. There are however disadvantages to their use: firstly, neither variables provide information about different sub-groups. Secondly, as “Country of Nationality” identifies the country where the patient is registered as citizen, it is not possible to distinguish country nationals born abroad. Thirdly, there is the risk of misalignment with population denominators published by Eurostat and UN-DESA, which only identify as migrants people with a history of regular migration of at least one year. Finally, ECDC [26] has identified low data completeness of both variables in TESSy databases (for “Country of Nationality” more than for “Country of Birth”). This limits their use because it leads to an under-attribution of infectious disease cases to migrant populations. The reasons for this low completeness have yet to be explored.

In EU/EEA surveillance, there is a lack of variables focusing on other migration characteristics, such as migrant status. This limits the possibility of stratifying data according to a known risk factor and of aligning surveillance data with population data available by migration status (from Eurostat, UN-DESA and UNHCR).

Data on behavioral factors and on socio-economic factors are also scarce. The variable “imported case” does not distinguish between cases in migrants and cases in non-migrant travellers and might be difficult to assess in the case of chronic diseases with long, latent asymptomatic phases such as TB, HIV and syphilis. This is particularly relevant for migrants with unknown health status upon arrival.

While demographic variables are present, current definitions do not align perfectly with Eurostat variable aggregations. Firstly because age is currently coded in surveillance with different age groups and secondly because the variable “sex” includes a “transgender” option that is not present in the Eurostat variable definition.

While it is unlikely that an extensive number of new variables can be included in routine surveillance of infectious diseases in the EU/EEA, this multi-dimensional framework could stimulate exchanges among Member States for increasing variable completeness and/or adding selected variables in EU/EEA surveillance. Furthermore, should the completeness of “Country of Birth” and “County of nationality” improve, these two variables could be used to identify three subpopulations under the domain “migration characteristics”, as shown in Table 4. A further distinction between reporting country nationals and other EU/EEA nationals, among people born outside the EU/EEA, was not proposed in Table 4 because it might lead to excessively small groupings.

**Table 4:** Migrant population subgroups diversified on the basis of existing variables.

Migrant Population Subgroups	Variable “Country of Birth”	Variable “Country of Nationality”
Intra EU mobile population *	EU/EEA country different from reporting country	EU/EEA country different from reporting country
EU/EEA first generation immigrants—non-nationals	Different from EU/EEA	Different from EU/EEA
Longer time EU/EEA resident first generation immigrants/immigrants born from EU/EEA citizens	Different from EU/EEA	EU/EEA country

* including second generation migrants born in EU/EEA countries granting citizenship by birth in the territory (*ius soli*). Granting citizenship to stateless people and foundlings born in the country is common to most EU/EEA countries. In addition to this, *ius soli* can be granted in several EU/EEA countries to people born in the country with foreign parents who have lived in the country for several years [57].

Alternative tools might also be considered to collect variables that are not possible to include in statutory surveillance systems. In particular, cross sectional population-based surveys could be performed to estimate an adjusted infectious disease prevalence estimate for target populations including migrants in order to assess the impact of disease. This multi-dimensional framework might be used to support discussions among Member States on how to conduct comparable national cross sectional surveys on aspects related to migrant health and infectious diseases in the EU/EEA. These surveys might be particularly useful in assessing the burden of infections with chronic latency periods among host and migrant populations. Such studies might also explore if differences exist in prevalence and disease burden in sub-population groups stratified according to the data collection domains proposed.

One limitation of our literature review study was to design a narrow search strategy with only two search strings and restricting the time frame and our search to English and French language articles using a single database (PubMed). This decision made the number of articles identified manageable for the reviewer assigned to this analysis. We are aware that this approach could have led to the exclusion of scientific articles not indexed in PubMed. We considered this not to hinder the aim of the review that was not to comprehensively assess literature in relation to an intervention, but rather to verify whether the proposed data collection domains were able to take into account major reported risk factors for infectious diseases in migrant populations.

Another possible limitation to consider is that the analysis of existing data sources could be limited by the fact that we assessed available denominator data from datasets that were publicly available. It is possible that additional datasets, and/or that disaggregation of data groupings present in available datasets, could be available in non-public environments. This could have led us to overestimate the risk of numerator/denominator misalignment.

## 5. Conclusions

We approached, through this study, three of the priorities identified by ECDC [26] (on the basis of previous WHO indications [58]) for designing a framework to monitor infectious diseases among migrant populations. This work highlighted existing knowledge gaps and next steps. From the perspective of EU/EEA epidemiological surveillance, further studies are needed at national level to explore the reasons for the under-reporting of variables such as “Country of Birth” and “Country of Nationality”. The possibility of including additional variables currently not collected in infectious disease surveillance but within the domains of the multi-dimensional framework, could also be explored. From the perspective of alternative data collection tools, consensus meetings involving disease and migration experts in Member States will be needed to design a set of core variables and indicators for the conduction of comparable national cross sectional surveys on aspects related to migrant health and infectious diseases. This could be a first step towards the definition of common study protocols with the aim of improving comparability of data on migrant health and infectious diseases across EU/EEA studies and ultimately better inform public health action.

## References

[1] De la Rica S., Glitz A., Ortega F. (2013). Immigration in Europe: Trends, Policies and Empirical Evidence. http://www.econ.upf.edu/~glitz/ImmigrationEurope2013.pdf.

[2] Napoli C., Riccardo F., Declich S., Dente M.G., Pompa M.G., Rizzo C., Rota M.C., Bella A., The National Working Group (2014). An early warning system based on syndromic surveillance to detect potential health emergencies among migrants: Results of a two-year experience in Italy. Int. J. Environ. Res. Public Health.

[3] International Organization for Migration (2014). Global Migration Trends: An Overview. http://missingmigrants.iom.int/sites/default/files/documents/Global_Migration_Trends_PDF_FinalVH_with%20References.pdf.

[4] UNHCR Global Report 2012—Europe Regional Summary. http://www.unhcr.org/51b1d6260.html.

[5] UNHCR Regional Operations Profile—Europe. http://www.unhcr.org/pages/4a02d9346.html.

[6] EUROSTAT Migration and Citizenship Data. http://ec.europa.eu/eurostat/web/population-demography-migration-projections/migration-and-citizenship-data.

[7] EUROSTAT Migration and Migrant Population Statistics. http://ec.europa.eu/eurostat/statistics-explained/index.php/Migration_and_migrant_population_statistics.

[8] Agreement on the European Economic Area OJ No L 1, 3.1.1994, p. 3; and EFTA States’ Official Gazettes. http://www.efta.int/sites/default/files/documents/legal-texts/eea/the-eea-agreement/Main%20Text%20of%20the%20Agreement/EEAagreement.pdf.

[9] Riccardo F., Napoli C., Bella A., Rizzo C., Rota M.C., Dente M.G., de Santis S., Declich S. (2011). Syndromic surveillance of epidemic-prone diseases in response to an influx of migrants from North Africa to Italy. Euro Surveill..

[10] Norredam M., Agyemang C., Hoejbjerg Hansen O.K., Petersen J.H., Byberg S., Krasnik A., Kunst A.E. (2014). Duration of residence and disease occurrence among refugees and family reunited immigrants: Test of the “healthy migrant effect” hypothesis. Trop. Med. Int. Health.

[11] Antiretroviral Therapy Cohort Collaboration (ART-CC) (2013). Influence of geographical origin and ethnicity on mortality in patients on antiretroviral therapy in Canada, Europe, and the United States. Clin. Infect. Dis..

[12] Solé-Auró A., Crimmins E.M. (2008). Health of immigrants in European countries. Int. Migr. Rev..

[13] HPA Presentation at the Workshop on Migrant Health and Infectious Diseases in the EU/EEA. http://ecdc.europa.eu/en/press/events/Documents/ECDC-INSA-UK-country-presentation.pdf.

[14] Consumers, Health and Food Executive Agency (2014). Action on Health Inequalities in the European Union. The EU Health Programme’s Contribution to Fostering Solidarity in Health and Reducing Health Inequalities in the European Union 2003–2013. http://ec.europa.eu/chafea/documents/health/health-inequality-brochure_en.pdf.

[15] Rechel B., Mladovsky P., Ingleby D., Mackenbach J.P., McKee M. (2013). Migration and health in an increasingly diverse Europe. Lancet.

[16] Marschall T., Kretzschmar M., Mangen M.J., Schalm S. (2008). High impact of migration on the prevalence of chronic hepatitis B in the Netherlands. Eur. J. Gastroenterol. Hepatol..

[17] Barnett E.D., Weld L.H., McCarthy A.E., So H., Walker P.F., Stauffer W., Cetron M., GeoSentinel Surveillance Network (2013). Spectrum of illness in international migrants seen at GeoSentinel clinics in 1997–2009, part 1: US-bound migrants evaluated by comprehensive protocol-based health assessment. Clin. Infect. Dis..

[18] Gushulak B.D., MacPherson D.W. (2004). Globalization of infectious diseases: The impact of migration. Clin. Infect. Dis..

[19] Regional Office for Europe of the World Health Organization (2004). The Vector-borne Human Infections of Europe: Their Distribution and Burden on Public Health.

[20] Vakali A., Patsoula E., Spanakos G., Danis K., Vassalou E., Tegos N., Economopoulou A., Baka A., Pavli A., Koutis C. (2012). Malaria in Greece, 1975 to 2010. Euro Surveill..

[21] Odolini S., Parola P., Gkrania-Klotsas E., Caumes E., Schlagenhauf P., López-Vélez R., Burchard G.D., Santos-O’Connor F., Weld L., von Sonnenburg F. (2012). Travel-related imported infections in Europe, EuroTravNet 2009. Clin. Microbiol. Infect..

[22] European Centre for Disease Prevention and Control (2013). Mission report Dengue outbreak in Madeira, Portugal.

[23] EC Legal Framework for Communicable Disease Surveillance. http://ecdc.europa.eu/en/activities/surveillance/Pages/legal_framework.aspx.

[24] Decision No 2119/98/EC of the European Parliament and of the Council of 24 September 1998 Setting up a Network for the Epidemiological Surveillance and Control of Communicable Diseases in the Community Official Journal L 268, 03/10/1998 P. 0001–0007. http://eur-lex.europa.eu/legal-content/EN/ALL/?uri=CELEX:31998D2119.

[25] Decision No 1082/2013/EU of the European Parliament and of the council of 22 October 2013 on Serious Cross-Border Threats to Health Official Journal of the European Union L.293. http://eur-lex.europa.eu/LexUriServ/LexUriServ.do?uri=OJ:L:2013:293:0001:0015:EN:PDF.

[26] European Centre for Disease Prevention and Control (2014). Assessing the Burden of Key Infectious Diseases Affecting Migrant Populations in the EU/EEA.

[27] Kehr J. (2012). Blind spots and adverse conditions of care: Screening migrants for tuberculosis in France and Germany. Sociol Health Illn..

[28] Boulogne R., Jougla E., Breem Y., Kunst A.E., Rey G. (2012). Mortality differences between foreign born and locally born population in France (2004–2007). Soc. Sci. Med..

[29] Fernandes A., Pereira Miguel J. (2009). Health and Migration in European Union: Better Health for all in an Inclusive Society.

[30] Transparent Reporting of Systematic Reviews and Meta-Analyses (PRISMA) Statement. http://www.prisma-statement.org/statement.htm.

[31] Simon F., Gautret P., Nicolas X., Ausset P., de Pina J.J., Demortiere E. (2013). Crossing the Gulf of Aden: Cutaneous infections in African migrant shipwreck survivors. Travel Med. Infect. Dis..

[32] Wagner K.S., Lawrence J., Anderson L., Yin Z., Delpech V., Chiodini P.L., Redman C., Jones J. (2014). Migrant health and infectious diseases in the UK: Findings from the last 10 years of surveillance. J. Public Health.

[33] Zammarchi L., Borchi B., Chiappini E., Galli L., Brogi M., Sterrantino G., Trotta M. (2012). Syphilis in pregnancy in Tuscany, description of a case series from a global health perspective. J. Matern. Fetal Neonatal Med..

[34] Rosales C., Ortega M.I., de Zapien J.G., Contreras Paniagua A.D., Zapien A., Ingram M., Aranda P. (2012). The US/Mexico Border: A binational approach to framing challenges and constructing solutions for improving farmworkers’ lives. Int. J. Environ. Res. Public Health.

[35] Jaeger F.N., Hossain M., Kiss L., Zimmerman C. (2012). The health of migrant children in Switzerland. Int. J. Public Health.

[36] Kamper-Jørgensen Z., Andersen A.B., Kok-Jensen A., Kamper-Jorgensen M., Bygbjerg I.C., Andersen P.H., Thomsen V.O., Lillebaek T. (2012). Migrant tuberculosis: The extent of transmission in a low burden country. BMC Infect. Dis..

[37] Norredam M., Olsbjerg M., Petersen J.H., Bygbjerg I., Krasnik A. (2012). Mortality from infectious diseases among refugees and immigrants compared to native Danes: A historical prospective cohort study. Trop. Med. Int. Health.

[38] Tafuri S., Martinelli D., Melpignano L., de Palma M., Quarto M., Prato R., Germinario C. (2011). Tuberculosis screening in migrant reception centers: Results of a 2009 Italian survey. Am. J. Infect. Control.

[39] Ott J.J., Paltiel A.M., Winkler V., Becher H. (2010). The impact of duration of residence on cause-specific mortality: A cohort study of migrants from the Former Soviet Union residing in Israel and Germany. Health Place.

[40] Suurmond J., Rupp I., Seeleman C., Goosen S., Stronks K. (2013). The first contacts between healthcare providers and newly-arrived asylum seekers: A qualitative study about which issues need to be addressed. Public Health.

[41] Stoffels K., Allix-Beguec C., Groenen G., Wanlin M., Berkvens D., Mathys V., Supply P., Fauville-Dufaux M. (2013). From multidrug- to extensively drug-resistant tuberculosis: Upward trends as seen from a 15-year nationwide study. PLoS ONE.

[42] Kan B., Kalin M., Bruchfeld J. (2013). Completing treatment for latent tuberculosis: Patient background matters. Int. J. Tuberc. Lung Dis..

[43] Nyiri P., Eling J. (2012). A specialist clinic for destitute asylum seekers and refugees in London. Br. J. Gen. Pract..

[44] Sarivalasis A., Zellweger J.P., Faouzi M., Daher O., Deslarzes C., Bodenmann P. (2012). Factors associated with latent tuberculosis among asylum seekers in Switzerland: A cross-sectional study in Vaud County. BMC Infect. Dis..

[45] Chai S.J., Davies-Cole J., Cookson S.T. (2013). Infectious disease burden and vaccination needs among asylees *versus* refugees, district of Columbia. Clin. Infect. Dis..

[46] Fenner L., Gagneux S., Janssens J.P., Fehr J., Cavassini M., Hoffmann M., Bernasconi E., Schrenzel J., Bodmer T., Böttger E.C. (2012). Tuberculosis in HIV-negative and HIV-infected patients in a low-incidence country: Clinical characteristics and treatment outcomes. PLoS ONE.

[47] Takla A., Barth A., Siedler A., Stocker P., Wichmann O., Delere Y. (2012). Measles outbreak in an asylum-seekers’ shelter in Germany: Comparison of the implemented with a hypothetical containment strategy. Epidemiol. Infect..

[48] De Valliere S., Cani N., Grossenbacher M., Puig F., Masserey E., Bodenmann P. (2011). Comparison of two strategies to prevent varicella outbreaks in housing facilities for asylum seekers. Int. J. Infect. Dis..

[49] Redman E.A., Reay H.J., Jones L., Roberts R.J. (2011). Self-reported health problems of asylum seekers and their understanding of the national health service: A pilot study. Public Health.

[50] Dudareva S., Barth A., Paeth K., Krenz-Weinreich A., Layer F., Delere Y., Eckmanns T. (2011). Cases of community-acquired meticillin-resistant Staphylococcus aureus in an asylum seekers centre in Germany, November 2010. Euro Surveill..

[51] Wickramage K., Galappaththy G.N. (2013). Malaria burden in irregular migrants returning to Sri Lanka from human smuggling operations in West Africa and implications for a country reaching malaria elimination. Trans. R. Soc. Trop. Med. Hyg..

[52] Wickramage K., Premaratne R.G., Peiris S.L., Mosca D. (2013). High attack rate for malaria through irregular migration routes to a country on verge of elimination. Malar. J..

[53] Kaoutar B., Mathieu-Zahzam L., Lebas J., Chauvin P. (2012). La santé des migrants consultant la policlinique Baudelaire de l’hôpital Saint-Antoine à Paris, France. Bull. Soc. Pathol. Exot..

[54] EUROSTAT Migration and Migrant Population Statistics. http://epp.eurostat.ec.europa.eu/statistics_explained/index.php/Migration_and_migrant_population_statistics#Methodology_.2F_Metadata.

[55] United Nations Population Division Department of Economic and Social Affairs (UNDESA) Population Division International Migration. http://www.un.org/en/development/desa/population/migration/data/estimates2/estimatesage.shtml.

[56] United Nations High Commissioner for Refugees (UNHCR) Statistics and Operational Data. http://www.unhcr.org/pages/4a013eb06.html.

[57] European University Institute EUDO Observatory on Citizenship Comparing Citizenship Laws: Mode A02a: Birth in Country (2nd Generation). http://eudo-citizenship.eu/databases/modes-of-acquisition?p=dataEUCIT&application=modesAcquisition&search=1&modeby=idmode&idmode=A02a.

[58] World Health Organization (2010). Health of Migrants—The Way Forward.

